# The symbiosis of contact force catheter use for hybrid ablation for atrial fibrillation

**DOI:** 10.1007/s12471-015-0729-y

**Published:** 2015-07-08

**Authors:** N. Kumar, L. Pison, P. Lozekoot, R. Choudhury, M. La Meir, S. Gelsomino, H. Crijns, J. Maessen

**Affiliations:** Department of Cardiology, Maastricht University Medical Centre and Cardiovascular Research Institute Maastricht (CARIM), P. Debyelaan 25, PO Box 5800, 6229 HX Maastricht, The Netherlands; Department of Cardiology, AZ Sint- Jan Brugge- Oostende AV, Brugge, Belgium; Department of Cardiac Surgery, Maastricht University Medical Centre and Cardiovascular Research Institute Maastricht (CARIM), Maastricht, The Netherlands

**Keywords:** Catheter ablation, Atrial fibrillation, Hybrid procedure, Contact force, Surgical ablation

## Abstract

**Objective:**

Reconduction across an ablation line is a common reason for arrhythmia recurrence over time. The hybrid procedure combines epicardial ablation of the pulmonary vein (PV) and creation of a box lesion with endocardial touch-ups for any electrical gaps. A high contact force (CF) between the ablation tip and cardiac tissue may increase the risk of thrombus formation, catheter tip charring, steam pop formation, and even cardiac perforation. CF monitoring is a significant new parameter for titration of the CF for creating an adequate lesion.

**Methods:**

Thirty-eight consecutive patients underwent epicardial ablation using bipolar radiofrequency devices. After checking electrical bidirectional block of the ablation lines, an endocardial CF catheter was used for further ablation (if needed) to complete the isolation of PVs, box lesion, cavotricuspid isthmus (CTI), and complex fractionated atrial electrograms (CFAE).

**Results:**

Endocardial touch-up was needed for 2 PVs (1.3 %) and 10 (26.3 %) box lesions. It was also used for the CTI line in 7 (18.4 %) patients, atrial tachycardia in 3 (7.9 %) patients, and additional CFAE ablation in 17 (44.7 %) patients. All 5 patients with arrhythmia recurrence had a mean CF < 10 g (*p* = 0.03). Procedure duration was significantly shorter in the CF group (223 ± 57 vs. 256 ± 60 min, *p* = 0.03) compared with control group.

**Conclusion:**

Use of CF catheters is safe, feasible, and complementary to a hybrid procedure setup for atrial fibrillation ablation. Its real-time monitoring may predict future arrhythmia recurrence, and decrease procedure time.

## Introduction

Atrial fibrillation (AF) is the most common arrhythmia globally affecting more than 20 million people and is a leading cause of stroke among people 65 years and older [1].

During catheter ablation for a cardiac arrhythmia, energy is delivered to areas of interest of the heart muscle that are causing the abnormal heart rhythm. Reconduction across an ablation line is one of the most frequent reasons for arrhythmia recurrence over time, e.g. pulmonary vein (PV), and linear lesions [2]. The hybrid procedure combines the epicardial ablation of PVs and creation of the box lesion by the cardiac surgeon and endocardial touch-ups (only if needed) to ensure ablation of any missed electrical gaps, by the electrophysiologist [3–5]. To create a transmural lesion, contact force (CF) between the ablation tip and cardiac tissue is a major determining parameter for radiofrequency catheters, thus its monitoring assumes significance [6]. Low CF may lead to an inadequate lesion. However, if it is very high, it may increase the risk of significant complications such as thrombus formation, catheter tip charring, steam pop formation and also cardiac perforation. To date CF catheters have never been used in a hybrid procedure setting for AF ablation.

To the best of our knowledge, this is the first article to study the safety and feasibility of using a CF catheter during the hybrid ablation of AF.

## Methods

### Study population

Thirty-eight patients with symptomatic AF underwent the hybrid procedure using the epicardial bipolar RF devices and an endocardial CF catheter. The definitions of paroxysmal, persistent, long-standing persistent, and permanent AF, success and failure of ablation, and follow-up monitoring were based on the consensus statement of the Heart Rhythm Society, the European Heart Rhythm Association, and European Cardiac Arrhythmia Society.

Selection criteria of AF patients for this procedure were previously failed catheter ablation, failure of at least one antiarrhythmic drug (AAD), left atrial volume ≥ 55 ml, or patient preference for a hybrid procedure instead of a percutaneous approach. Prior catheter ablation was noted in the history for 18/38 AF patients (47.4 %) and 11/38 patients (29 %) had prior catheter ablation for cavo-tricuspid isthmus (CTI) dependent atrial flutter. The preoperative work-up consisted of transthoracic echocardiography, cardiac computed tomography (for assessing PV anatomy and integration to electroanatomical mapping), and pulmonary function testing.

### Control group

A random, retrospective and randomised control group of 30 patients with similar patient characteristics (Table 1) was selected, to compare the endocardial ablation parameters, e.g. fluoroscopy time, procedure time and ablation time with a similar ablation setting of the radiofrequency generator. A standard non-force sensing irrigated tip catheter (ThermoCool, Biosense Webster) was used during a hybrid procedure for the patients in the control group.

**Table 1:** Patient characteristics

Variables		Number
Patients (*n*)		38
Gender (men/women)		34/4
Age (years)		61.8 ± 7.2
BMI (kg/m^2^)		28.2 ± 3.4
Tested pulmonary veins (*n*, %)		150(100)
Touch up needed in pulmonary veins (*n*, %)		2 (1.3)
Tested box lesions (*n*, %)		37 (97.4)
Touch up needed in box (*n*, %)		10 (27 )
Additional CTI ablation (*n*, %)		7 (18.4)
Additional atrial tachycardia ablation (*n*, %)		3 (7.9)
Additional mitral line ablation (*n*, %)		0 (0)
Additional CFAE ablation (*n*, %)		17 (44.7)
Average procedure time (minutes)		223 ± 57
**Medical history**		
Hypertension (*n*, %)		16 (42.1)
Diabetes (*n*, %)		5 (13.2)
CAD (*n*, %)		15 (39.5)
COPD (*n*, %)		2 (5.3)
**Medication use**		
Amiodarone (*n*, %)		5 (13.2)
Flecainide (*n*, %)		19 (50)
Sotalol (*n*, %)		8 (21.0 )
**Disease characteristics**		
Paroxysmal/persistent/permanent AF (*n*/*n*/*n*, %/%/%)		14,19,5 (37,50,13)
AF duration (years, mean ± SD)		6.8 ± 4.352 ± 14
LVEF (%, mean ± SD)		93 ± 25
Volume (ml, mean ± SD) of prior catheter ablations (*n*, %)		18 (47.4)
Number of prior electrical/pharmacological cardioversions (*n*, %)		26 (68.4 )

*AF* atrial fibrillation, *AFl* atrial flutter, *BMI* body mass index, *n* numbers, *CTI* cavo tricuspid isthmus, *CAD* coronary artery disease, *CFAE* complex fractionated atrial electrograms, *CHF* congestive heart failure, *LVEF* left ventricle ejection fraction, *LA* left atrial, *SD* standard deviation

### Hybrid procedure

The hybrid procedure was performed as described elsewhere [3, 4, 7]. Briefly, all AADs were stopped before the procedure (considering their half-life) except amiodarone. Under general anaesthesia, after introducing two 5 mm working ports and a 5 mm video port in the intercostal spaces of the left and right hemithorax, patients underwent PV isolation using epicardial bipolar radiofrequency clamps (Atricure, West Chester, OH, USA). An epicardial box lesion was made using a bipolar radiofrequency pen or a linear pen device (Coolrail, Atricure) consisting of a roof line (connecting both superior PVs) and an inferior line (connecting both inferior PVs). Using left femoral vein access, catheters were placed in the His bundle and coronary sinus. Using a single transseptal puncture a circular mapping catheter, according to the size of the PV (Lasso, Biosense Webster Inc., Diamond Bar, USA), was placed ostially to assess the PV potentials. The electrophysiologist checked for electrical isolation of ablation lines by pacing manoeuvres, electroanatomical mapping and/or adenosine bolus administration. A CARTO platform (CARTO-3; Biosense Webster, Diamond Bar, CA) was used for electroanatomical mapping. A left atrium shell was created using point-by-point acquisition with the aid of the CF catheter (SmartTouch^TM^, Biosense Webster Inc.). Criteria for an adequate left atrial shell were: ≥ 100 points, which were homogenously distributed to create the entire chamber.

**Fig. 1:**
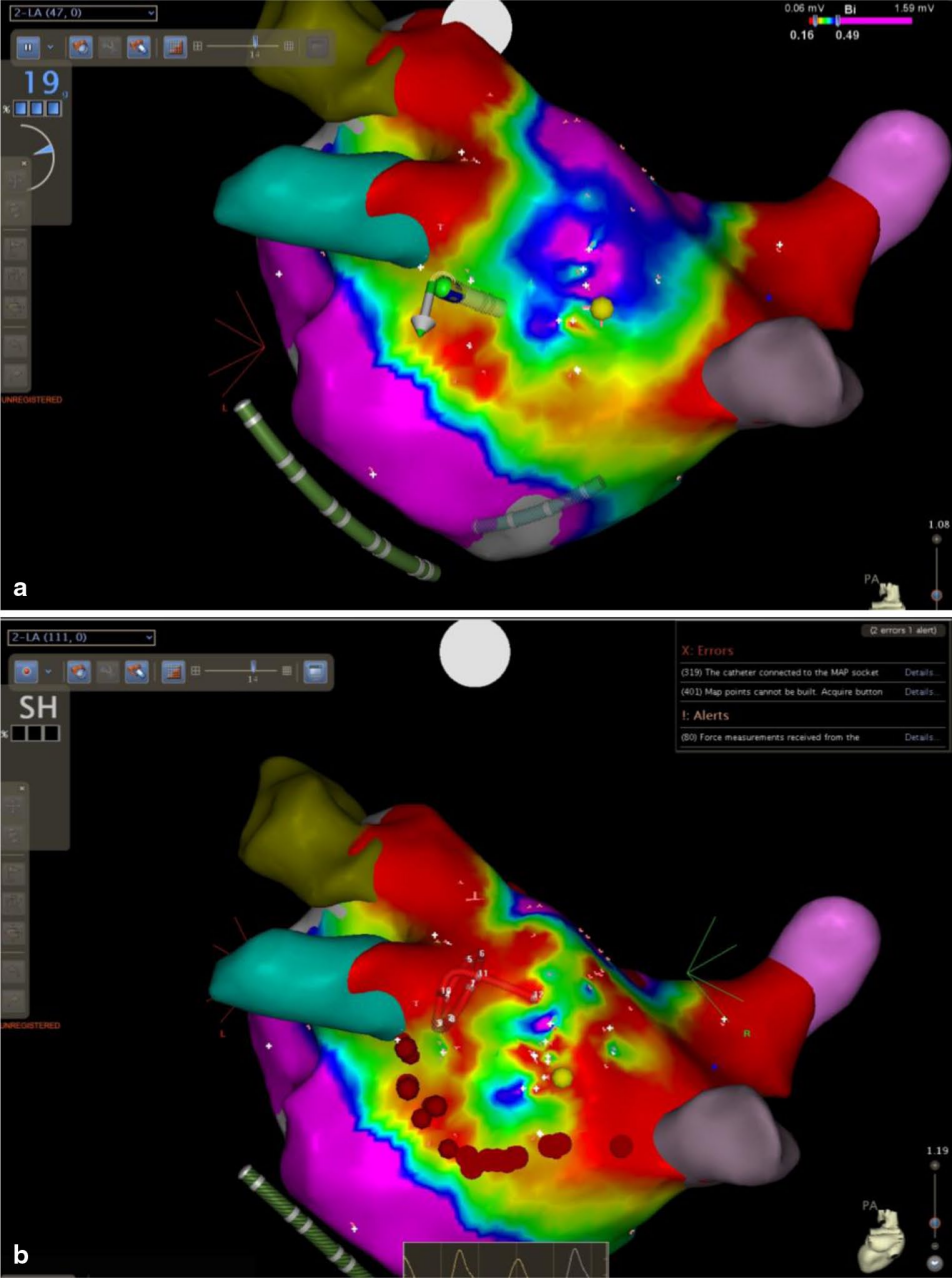
Endocardial radiofrequency ablation (*red dots*) using CF catheter for completion of the inferior line of box lesion. **a** At start of ablation. **b** After finishing ablation. We can further notice the visual change in the voltage map after finishing the ablation

The CF catheter was further used for endocardial ablation (if needed) to complete the PV isolation and box lesion, while maintaining the CF values between 10–40 G with the aid of a Stockert 70 radiofrequency generator (Biosense Webster) with a maximal temperature of 43 °C and limited to 25 W at the posterior wall (Fig. 1). Endocardial ablation was also used for CTI ablation in patients with a prior history of atrial flutter, any inducible atrial tachycardia, and complex fractionated atrial electrogram (CFAE) ablation is used in case of persistent AF after epicardial PV isolation and box lesion. The spatial distribution of CFAE in the left atria of AF patients was evaluated using software designed to automatically identify CFAE in the framework of the CARTO platform. In detail, the minimal amplitude threshold and maximal amplitude threshold were set to ± 0.05 millivolt (mV) and ± 0.15 mV, and the minimal and maximal intervals between two consecutive peaks were set to 60 msec and 120 msec [8].

After finishing the endocardial ablation, and retesting for arrhythmia, the induction was performed. The left atrial appendage (LAA) was removed using a stapling device in 11 patients. Finally, bilateral chest drains were inserted in the pleural cavities. Patients were extubated in the operating room before being transferred to the ICU. Within 6 h, low-molecular-weight heparin was started and acenocoumarol was restarted after 2 days. AADs were restarted as soon as possible. After confirming absence of atrial arrhythmias after 6 months, AADs and anticoagulation were discontinued.

### Follow-up

Follow-up was conducted at our hospitalʼs outpatient clinic in the 3rd, 6th, 9th, 12th month after the procedure. A 7-day Holter monitoring was used for all patients; if not available patients underwent at least 48-hour Holter monitoring (3 patients). Arrhythmia recurrence was considered to be any episode of AF, flutter or tachycardia lasting more than 30 s detected after the 3-month blanking period.

### Statistical analysis

Continuous variables are expressed as mean ± standard deviation. Data were retrospectively entered into a database. Statistical analysis was performed using SPSS 16.0 (SPSS Inc, Chicago, IL). Event-free survival was estimated using Kaplan-Meier and compared by the log-rank test. Possible predictors of arrhythmia recurrence were performed using the Cox proportional hazards regression models. A *p* value of < 0.05 was considered significant.

## Results

The study group consisted of 38 patients (61.8 ± 7.2 years; 34 male) with AF duration of 6.8 ± 4.3 years. A left-sided common ostium was observed in 2 patients. The body mass index was 28.2 ± 3.4 kg/m^2^. Eighteen patients had undergone catheter PV isolation. Electrical/pharmacological cardioversions were attempted previously for 26 patients (68.4 %). The mean procedure time (from initial skin incision to skin closure) was 223 ± 57 min. The mean left atrium size was 93 ± 25 ml, with a mean left ventricular ejection fraction of 52 ± 14 %. Epicardial bipolar radiofrequency devices were used for 150 PVs (100 %) and 37 box lesions (100 %) for 38 patients. Endocardial touch-up using a CF catheter was needed for 2 PVs (1.3 %) of different patients, i.e. left superior PV and right inferior PV, and for 10 box lesions (26.3 %). A total of 4 touch-up lesions with a mean duration of 39 s were applied to the PVs. It was also used for creation of a CTI line in 7 (18.4 %) patients and additional CFAE ablation in 17 (44.7 %) patients. Further, during burst pacing to test for inducible AF, 3 (7.9 %) patients developed 5 different morphologies of inducible atrial tachycardia and henceforth CF was also used for endocardial ablation for all of them. The mean grams of force per ablation lesion were 17.6 ± 7.8 g for the right PVs and 15.7 ± 7.2 g for the left PVs. Further details on the lesion sets and patient characteristics are shown in Table 1.

The control group consisted of 30 patients (paroxysmal: 11 (37 %), persistent: 16 (53 %), permanent = 3 (10 %)). The mean left atrial volume was 91 ± 23 ml. The statistical analysis (using the Mann–Whitney test) for procedure duration was significantly shorter in the CF group (223 ± 57 vs. 256 ± 60 min, *p* = 0.03) compared with the control group. There were no statistically significant differences observed in ablation time (11.2 ± 5.9 vs 14.1 ± 6.2 min) or fluoroscopy time (18.5 ± 8.6 vs 23.4 ± 6.4 min) although all were reduced in the CF group. No difference in complications was seen between the groups. After a mean follow-up of 357 ± 43 days (395–334 days) for control group patients, 75 % of patients were AF free without AADs.

## Follow-up

During the first 3 months after the procedure, the patients were blanked for the analysis of recurrence of AF. After follow-up of 335 ± 66.5 days (410–320 days), 33/38 patients (86.8 %) of the study population were in sinus rhythm without AADs, 2 patients needed AADs to maintain sinus rhythm. Catheter ablation was needed for 3 patients i.e. 1 typical CTI dependent atrial flutter, 1 mitral isthmus dependent flutter, and an atrial tachycardia. Non-inducible patients, who were tested immediately after ablations, had less arrhythmia during follow-up than inducible ones (*p* = 0.04). Among all patients with recurring arrhythmia, we retrospectively observed that they had received endocardial touch-up; the mean CF was < 10 g (*p* = 0.03), in spite of continuous attempts to improve the contact. Figure 3 shows a Kaplan-Meier graph of AF-free survival.

**Fig. 2:**
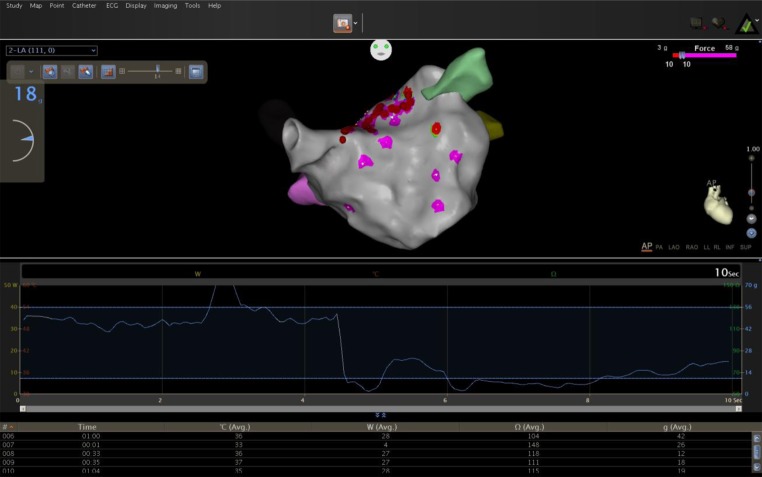
Temporal display of change of contact force (grey colored) with time during endocardial radiofrequency ablation (red dots)

## Adverse events

No thromboembolic complications or conversion to cardiopulmonary bypass were encountered. No signs of infection and phrenic nerve paralysis were seen among any of the patients. One patient had recurrent pain in the left thorax region a few weeks after the procedure. Diagnosis of atraumatic lung hernia was confirmed by high-resolution computerised tomographic (HRCT) [9, 10]. Retrospectively, the aetiology was attributed to widening of the mini invasions of 12 mms (initially) to 3 cm to accommodate an epicardial left atrial appendage (LAA) clip. To the best of our knowledge, this is the only case of atraumatic lung hernia secondary to usage of an LAA clip in the literature. No other complications were associated with such clip usage. No signs of PV stenosis were seen in any patient during follow-up HRCT [11].

## Discussion

This manuscript describes, for the first time, combined simultaneous thoracoscopic epicardial ablation using bipolar devices and endocardial ablation using CF catheters for paroxysmal and persistent AF. Further, the use of a CF catheter results in superior transmurality of linear ablation lesions.

The pharmacological approach with AADs has not achieved optimal results in the long-term maintenance of sinus rhythm in patients with AF [12−14]. Considering the limitations of medical therapy, there has been an upsurge in catheter and surgical ablation techniques for AF ablation [15−17]. The recently proposed hybrid approach combines the advantages of individual approaches while simultaneously doing away with their disadvantages, complications, and ultimately ensuring the transmurality of lesions [3, 5, 18]. AF recurrence is often associated with PV reconnection after initial PV ablation [19−23]. It is attributed to the inability of endocardial catheter based ablation techniques to guarantee long-lasting transmurality of the lesions [2].

Both ablation technologies used in this setup share common principles. An epicardial bipolar radiofrequency device (Atricure Inc.) works on the principle of impedance change and endocardial ablation using a CF catheter works on CF measurement. Interestingly, several studies have correlated impedance drop with CF with due credit to the more detailed quantitative analysis and use of nonparametric statistics [24−26]. Martinek et al. found that the use of CF sensing technology catheters significantly reduces ablation time, translating to an overall reduction in procedural times in PV isolation. This reasoning is well suited to the hybrid procedure as well. Avoiding radiofrequency delivery in areas with insufficient tissue electrode contact substantially reduced energy delivery. These catheters also significantly reduced ablation and procedural times in PV isolation [6]. Continuous monitoring of CF values may help to provide extra helpful information to the operating physician for safer catheter manipulation. It helps to make an AF ablation procedure safer as high CF values may occur, not only during catheter manipulation but also during ablation (Fig. 2 and video 1, [27]).

**Fig. 3:**
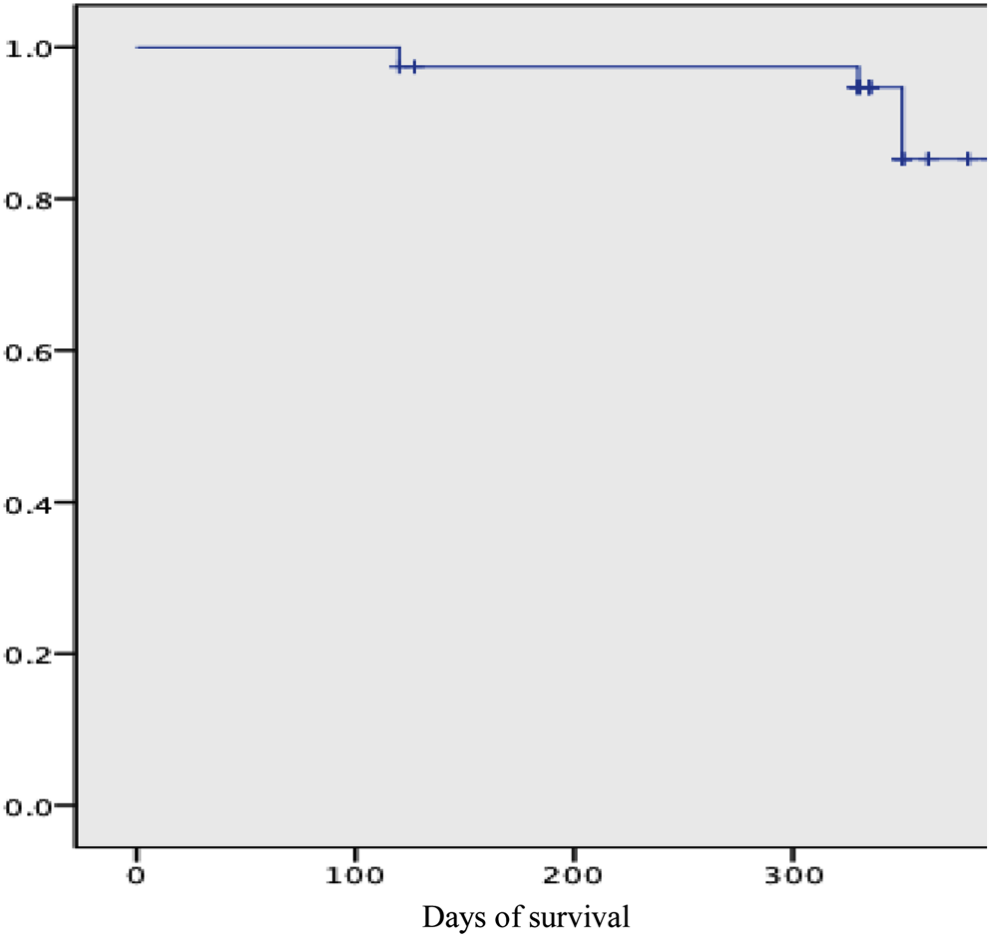
Kaplan–Meier survival curve of atrial arrythmia free survival after a single hybrid procedure after a blanking period of 90 days for the arrhythmias. (DAYS= follow up in days)

Higher incidence of cardiac tamponade is seen with endocardial left atrial linear ablations [28]. When endocardial tissue is exposed to high levels of radiofrequency energy, it may lead to cardiac tamponade with steam pop [29]. Other than radiofrequency energy, CF is a significant cofactor for cardiac rupture and thrombus. Several preclinical studies have associated it with significant increases in radiofrequency lesion depth, diameter, and volume [30−33].

Non-inducible patients tested immediately after ablations (using adenosine bolus, burst pacing and electrical testing) had less arrhythmias during follow-up than inducible ones [34]. This further signifies the importance of testing protocols used at the end of procedures. Lesion size is significantly affected by CF, e.g. lower radiofrequency power (30 W) and high CF (30–40 g) produces larger and deeper lesions while compared with the ones produced at greater power (50 W) but lower CF. Different anatomical sites in the left atrium can be difficult to approach with a catheter. Thus continuous measurement of catheter—tissue CF during radiofrequency ablation assumes significance to achieve good quality lesions [35].

There was significantly less need for endocardial touch-ups for epicardial ablation of PVs compared with other epicardial ablations, which again reminds us of the robustness of epicardial clamps compared with the epicardial pen used for box lesions. Procedural duration and ablation efficacy are significantly determined by catheter–tissue CF. Moreover, the number of lesions with low average CF or force time integral (FTI) and the time to achieve acute PV isolation are linearly related [36, 37]. Acute PV reconnection has been strongly associated with low values of CF and FTI [33]. In our study too, reconduction zones were localised in areas with mean CF readings < 10 g, in spite of continuous attempts to improve the contact. Low CF can predict gap formation in ablation FTI [35, 37] Moreover, Kumar et al. reported that optimal CF might be easier to obtain and maintain during right as compared with left PV ablation [35]. In our study too, the time with poor CF values (5 g) was significantly shorter for right than for left PV ablation [38].

The LAA was not excluded among the remaining patients due to anatomical restrictions or surgeon preference. LAA exclusion may eliminate the extra PV triggers of AF, and might also further decrease stroke incidence in patients with a high CHA2DS2-VASc score [39, 40] but can also cause bleeding [41], which might require conversion to sternotomy. Its effect on left atrial function and remaining thromboembolic risk is incompletely understood [42]. LAA removal has not always been associated with a higher success rate [43]. So, in future as more patients are receiving such LAA exclusion therapies, its role to determine the success of an AF ablation will be worth looking for.

The strength of this study lies in its exclusive approach to AF ablation and good follow-up. Further, this procedure is less invasive with minimum complications, unlike the pure surgical approach. The wastage of unnecessary energy delivery with CF catheters was avoided in three way: firstly by simultaneous epicardial ablation, secondly by visualising the electrical gaps by pacing manoeuvres and electroanatomical mapping and, finally by avoiding ablations in locations with insufficient surface contact. Moreover, the success rate of this procedure is comparable with either approach individually, especially considering the dilated left atrium and long years of AF duration. Monitoring of CF values during ablation can control the success rate of the procedure.

## Study limitations

The small number of patients and a control group in this single-centre retrospective study prevents definitive conclusions. However, future larger, long-term and multicentre studies may corroborate our results.

## Conclusion

Use of CF catheters is safe, feasible, and complementary to a hybrid procedure setup for AF ablation. The monitoring of real-time CF values predicts future arrhythmia recurrence and decreases procedure time.
